# Real-Time High-Performance Laser Welding Defect Detection by Combining ACGAN-Based Data Enhancement and Multi-Model Fusion

**DOI:** 10.3390/s21217304

**Published:** 2021-11-02

**Authors:** Kui Fan, Peng Peng, Hongping Zhou, Lulu Wang, Zhongyi Guo

**Affiliations:** 1 School of Computer and Information, Hefei University of Technology, Hefei 230009, China; fankuihfut@163.com (K.F.); pengpeng2021m@163.com (P.P.); 2 Biomedical Device Innovation Center, Shenzhen Technology University, Shenzhen 518118, China; wanglulu@sztu.edu.cn

**Keywords:** defect detection, ACGAN, sample generation, multi-algorithm model fusion

## Abstract

Most of the existing laser welding process monitoring technologies focus on the detection of post-engineering defects, but in the mass production of electronic equipment, such as laser welding metal plates, the real-time identification of defect detection has more important practical significance. The data set of laser welding process is often difficult to build and there is not enough experimental data, which hinder the applications of the data-driven laser welding defect detection method. In this paper, an intelligent welding defect diagnosis method based on auxiliary classifier generative adversarial networks (ACGAN) has been proposed. Firstly, a ten-class dataset consisting of 6467 samples, was constructed, which originate from the optical and thermal sensory parameters in the welding process. A new structured ACGAN network model is proposed to generate fake data similar to the true defect feature distributions. In addition, in order to make the difference between different defects categories more obvious after data expansion, a data filtering and data purification scheme was proposed based on ensemble learning and an SVM (support vector machine), which is used to filter the bad generated data. In the experiments, the classification accuracy can reach 96.83% and 85.13%, for the CNN (convolutional neural network) algorithm model and ACGAN model, respectively. However, the accuracy can further improve to 97.86% and 98.37% for the fusion models of ACGAN-CNN and ACGAN-SVM-CNN models, respectively. The results show that ACGAN can not only be used as an algorithm model for classification, but also be used to achieve superior real-time classification and recognition through data enhancement and multi-model fusion.

## 1. Introduction

In the manufacturing process of metal products, laser welding, as an important processing technology, has been widely used in many fields of industry. In the laser welding process, there will be severe thermal conversion effects, which require extremely high precision for the welding process parameter and fixing workpiece, and small weld deviation can lead to serious welding defects, which affects the quality of the welded products [1]. Moreover, the difference of welding materials and welded joints will also affect the quality of welding [2,3,4]. Therefore, rapid and effective defect detection of welded products is particularly important in the process of mass production. Existing laser welding defect detection schemes are mainly focused on the detection of post-welding defects. Conventional nondestructive testing methods are widely used but they have some limitations [5,6,7]. For example, visual testing can easily miss detection [8,9,10]; radiographic testing has radiation risk and its equipment cost is high [11]; magnetic particle testing and penetration testing can locate the defect position but cannot specifically display its shape; and eddy current detection and other signal detection technologies can not directly reflect the defect shape [12].

Because the actual process of production and manufacturing is complex and changeable, different instruments and methods are needed during defect detection in different processes. In most cases, a single device or method cannot accurately complete the detection task, however, it is impossible to integrate all the corresponding instruments and methods into one single system due to the large workload and high complexity of detecting every possible problem in each production process [13]. In the process of laser welding, the above-mentioned problems still exist. Thus, due to the complexity of both processes and defects, the judgement system is difficult to establish.

In the existing researches, four kinds of sensors are mainly used to monitor the laser welding process, such as visual [14,15,16], acoustic [17,18,19], optical [20,21] and thermal [22,23] sensors. These sensors are used to capture the changes of characteristic quantities during the welding process [24,25,26,27] and establish a correlation between the changing trends of characteristic quantities and the final welding effect. In the actual production line, it is difficult to realize real-time visual testing because of the need to capture and process a large amount of image data. In addition, the strong light during laser welding can lead to image saturation and thus reduce the accuracy of the system. Moreover, due to the unavoidable background noise in the production line, it is not suitable to integrate acoustic sensors into the laser welding process monitoring system to detect defects.

With the rapid developments and maturity of deep learning theory, intelligent fault diagnosis technology has become a research hotspot and development direction in the field of artificial intelligence. At present, the intelligent fault diagnosis algorithms mainly include artificial neural network [28,29,30,31], fuzzy logic [32,33], etc., which developed from single strategy classification prediction to multi-strategy fusion [34,35,36]. In addition, in some actual experiments, it is found that sample data is difficult to obtain, so that it is difficult for the deep learning method to fully learn the differences between different categories of data. To solve this problem, a data enhancement method is commonly used to expand the training data set. In 2020, Bal et al. [37] explored an effective machine learning method, the extreme learning machine, to predict the number of software faults, which could predict the software fault type in time when the software fault data was unbalanced. Generative adversarial networks (GAN) proposed by Goodfellow et al. [38] have been widely used in image processing and natural language processing, in which the generator and discriminator compete with each other, and fake data obtained from the generator and real data can be used to train the discriminator. By adversarial learning mechanism and adding new samples, the discriminator ability and the generation ability can be improved simultaneously, so as to improve the learning and generalization ability of the neural network. In 2020, Waheed et al. [39] proposed Covid-GAN which can produce composite images and can be used to enhance CNN (convolutional neural network) detection performance. In a brain computer interface (BCI) system, the performance of a classifier depends on the quality and quantity of training data to a great extent. Fahimi et al. [40] proposed a framework based on the deep convolutional generative adversarial networks for generating artificial electroencephalogram to augment the training set in order to improve the performance of a BCI classifier. In 2021, Guo et al. [41] developed a welding defect detection method using a generative adversarial network combined with transfer learning which is proposed to solve the data imbalance and improve the accuracy of defect detection. Jiang et al. [42] proposed a data selection strategy based on data filtering and data purification in model training, which combines supervised learning and the data generation process to obtain an end-to-end model.

The main contributions of this paper are summarized as follows.

(1) A hybrid welding fault diagnosis scheme based on ACGAN [43,44] (auxiliary classifier generative adversarial networks) and CNN [45,46] model has been proposed. Fake data are generated by the ACGAN generator using real data, and the CNN classifier is trained with both fake data and real data. Test samples are then input into the trained CNN model for fault diagnosis and prediction.
(2) Secondly, in order to increase the difference between categories and improve the recognition performance of the classifier, a data filtering and purification scheme based on ensemble learning is proposed. Multiple support vector machines (SVMs) [47,48] are used to learn different features of defect states and make integrated classification judgments. This integrated classifier filters out the bad data generated by the generator. The filtered data and the original training data are then put into the CNN model for training.
(3) Finally, under different amounts of training data, the ability of different models to identify welding defects is tested. Through experimental comparison with other classical classification models, the superiority of the ACGAN-SVM-CNN detection scheme has been proved.

The rest of this paper is organized as follows. Section 2 briefly introduces the basic theory of ACGAN. Section 3 describes the proposed hybrid detection approach, and the effectiveness and superiority of the proposed method have been investigated and proved by the comparative experiments of different models with different amounts of original data. Finally, conclusions are drawn in Section 4.

## 2. Auxiliary Classifier Generative Adversarial Networks (ACGAN)

GAN is an unsupervised deep learning model, from which other types of network structures can be derived, such as conditional generative adversarial nets (CGAN) and semi-supervised learning with generative adversarial networks (SGAN). CGAN improves the quality of generated data by combining tag information, while SGAN improves the data quality by reconstructing tag information. On the basis of these two networks, ACGAN extends its advantages and adds a category classification network to the system.

### 2.1. Structure Principle

**Figure 1:**
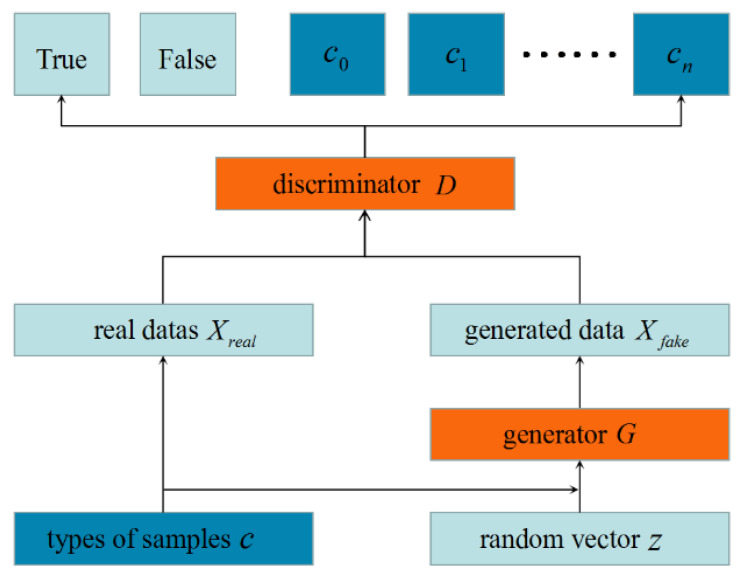
Main Structure of ACGAN.

The ACGAN is improved by the GAN model with supervised mechanism. The main difference between ACGAN and GAN is the added label information of auxiliary training. As shown in Figure 1, ACGAN is composed of a generator and a discriminator, whose output contains judgment information of not only the true or false data, but also the data category. Meanwhile, ACGAN considers the diversity of samples better than other varieties of GAN models.

ACGAN can generate higher quality samples using auxiliary classification tags. The ‘c’ in Figure 1 represents the class label of the corresponding data. The loss function of AGAN contains two parts, as shown in Equations (1) and (2), respectively.

(1) $$L_{s}=E_{x∼P_{data}}\left[\log D\left(x\right)\right]+E_{z∼P_{z}}\left[\log\left(1-D\left(G\left(z\right)\right)\right)\right]$$

(2) $$L_{c}=E_{c∼P_{data}}\left[\log D\left(c\right)\right]+E_{c∼P_{z}}\left[\log\left(1-D\left(G\left(c\right)\right)\right)\right]$$

In the above formulas, $L_{s}$ represents the cost function for data authenticity and $L_{c}$ represents the cost function for data classification accuracy. E represents the operation to find the mathematical expectation, $x/c∼P_{data}$ ensures x/c obeys the original data distribution, $z/c∼P_{z}$ ensures z/c obeys the Gaussian distribution and z represents random noise. Since the discriminator should distinguish the generated data from the real data as much as possible, for classifying the data effectively, the training goal of discriminator D is to make $L_{c}+L_{s}$ as maximum as possible. Meanwhile, it is expected that the data generated by the generator will be recognized as real data by the discriminator and effectively classified, which means that the goal of generator G training is to make $L_{c}-L_{s}$ as large as possible.

### 2.2. The ACGAN Training Process

The training process of ACGAN is basically the same as that of GAN. Based on the theory of zero-sum game, the discriminator and generator are trained alternately to achieve the final optimization effect.

In the ACGAN model, the first step is to train the discriminator with real data and fake data generated by the generator. When the discriminator training is completed, the parameters of the discriminator can be kept unchanged temporarily, at which the parameters of the discriminator will not be updated, and only the parameters in the generator will be updated according to the loss of feedback from the discriminator. The generator will generate fake data which is closer to the real data distribution. The updated generator will generate a new generation of fake data, and then train the discriminator with the new generation of fake data and real data. The iteration training repeats as above. The specific training steps are described as follows:

Input the randomly generated noise z vector with Gaussian normal distribution into generator G, and then generate fake data G(z).

Mark the fake data $X_{fake}=G(z)$ generated by generator G as 0 and the corresponding category label c is attached. Real data $X_{real}$ is marked as 1 and the corresponding category label c is also attached. The real and fake data are input into discriminator D together in batches, and the network terminal outputs the distinguished result through softmax classifier. The objective function of the optimized discriminator is as follows:

(3) $$L_{D}=\log B_{real}+\log(1-B_{fake})+\log C_{real}+\log C_{fake}$$

In Equation (3), $C_{real}$ is the category probability of multi-classification output when real data are input into the discriminator, and $C_{fake}$ is the category probability of multi-classification output when fake data are input into the discriminator. $B_{fake}$ is the real/fake (1/0) binary output when the fake data are input into the discriminator, and $B_{real}$ is the real/fake (1/0) binary output when the real data are input into the discriminator.

Keep the parameters of discriminator D unchanged, input the random noise vector z into generator G to generate fake data $X_{fake}=G(z)$, and attach the corresponding category label c. Fake data and real data are labeled as 1 together and input to discriminator D. When discriminator D determines that fake data $X_{fake}$ is false (the output label is 0), it means that the fake data $X_{fake}$ fails to deceive discriminator D successfully. In order to make the fake data generated by generator G successfully deceive discriminator D, it is necessary to maximize the objective function $L_{G}$ of the generator. The definition of $L_{G}$ can be expressed as follow.

(4) $$L_{G}=\log B_{fake}+\log C_{real}+\log C_{fake}$$

Repeat steps (1)–(3) to train the discriminator D and generator G iteratively until Nash equilibrium is reached, in which the true and fake resolution of the discriminator D are 50%. This indicates that the effect of the data generated by the generator is closest to the distribution of the original data, and the trained discriminator can be used for defect detection and classification.

## 3. Algorithmic Design and Experimental Analysis

In this section, we describe the ACGAN structure in detail and propose a data filtering and data purification strategy based on ensemble learning. By comparing different models and training data settings with different amounts of data, the superiority of the proposed welding defect detection scheme is demonstrated.

### 3.1. Data Acquirement Method and Data Description

**Figure 2:**
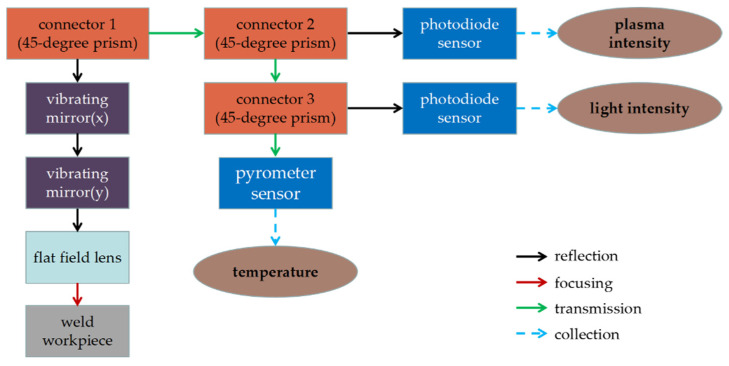
Structure diagram of data acquisition system.

The scanning laser welding machine mainly includes an SPI (Southampton Photonics Inc., Southampton, UK) pulsed fiber laser, a galvanometer scanner, a flat field lens and two vibrating mirrors. The galvanometer scanner can reflect laser light to the desired position by turning the vibrating mirrors to change the laser path. The front-end signal acquisition consists of two different photodiode sensors, a thermometer sensor and some optical elements. The overall schematic structure of the data acquisition system is shown in Figure 2. The first photodiode sensor is installed behind a 45-degree prism to obtain plasma intensity. After the other 45-degree prism, the second photodiode sensor is set up to capture light intensity information. Pyrometer sensors at the end can monitor temperature changes during welding.

In the process of the data collection experiment, 130 sampling times are set for each standard workpiece welding process, and the values of plasma intensity, light intensity and temperature are collected at each sampling time. In this way, we collected and constructed a data set with 6467 data, which contains 10 welding defects. The standard part is SUS304 stainless steel plate (Jiangsu Weigang alloy products Co., Ltd., Wuxi, China) with the thickness of about 0.3 mm and a radius of 22 mm. CW (continuous wave) optical fiber laser (Suzhou Chuangxuan Laser Technology Co., Ltd., Suzhou, China) was used in the experiment. The laser output power is 80 W, and the welding speed is 50 mm/s.

**Figure 3:**
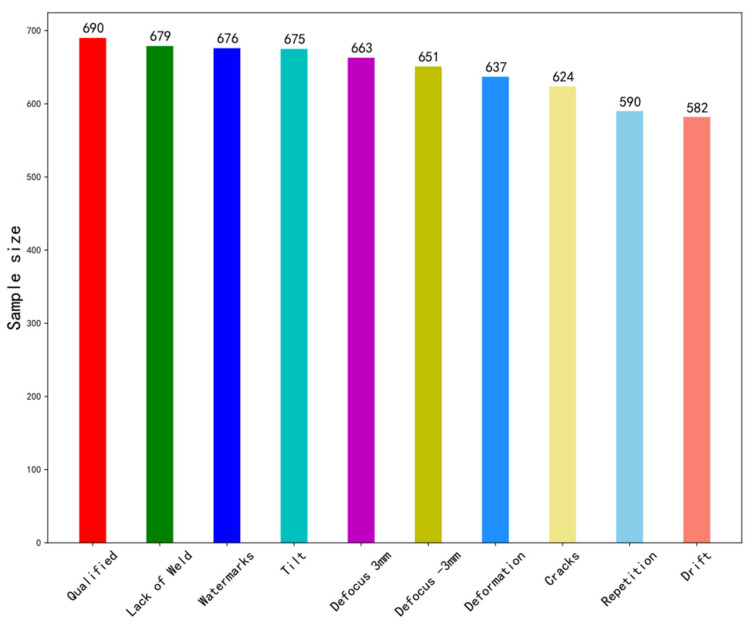
The composition of the data set.

After the data are collected, they need to be standardized. Here we convert the data to a standard dataset with a mean of 0 and a variance of 1. The ten kinds of welding defects are “Qualified”, “Defocus 3 mm”, “Defocus −3 mm”, “Deformation”, “Cracks”, “Repetition”, “Lack of Weld”, “Drift”, “Tilt” and “Watermarks”, respectively. Additionally, the concrete data amounts have been shown in Figure 3. In order to avoid the mode collapse during training, we try to keep the same amount of data for each category when collecting sample data. The descriptions of these categories are as follows:

1. “Qualified” means that no weld defects have been found and that they meet the technological requirements.
2. “Defocus 3 mm” refers to the defocusing distance over 3 mm. The focus plane above the workpiece is positive defocus, while the focus plane below the workpiece is negative defocus. The defocusing distance of excessively large absolute value leads to the overly low power density acting on the workpiece, making it difficult to reach the purpose of welding.
3. “Defocus −3 mm” represents defocusing distances of less than −3 mm.
4. “Deformation” means that as the welding current increases, the width of the weld increases, and splashes occur gradually, resulting in oxidative deformation and roughness on the surface of the weld product.
5. “Cracks” refer to high temperature cracks. In the process of laser welding, due to the small heat input of laser, the welding deformation and welding stress are small, thus generally, high temperature cracks will not occur.
6. “Repetition” means to weld again based on the existing welded seam.
7. “Lack of Weld” indicates that there are some missing welding points. ‘Lack of Weld’ is a widespread operation error.
8. “Drift” indicates the welding position suddenly drifted.
9. “Tilt” represents the base metal’s tilt during welding, so that defocusing distance has been changed.
10. “Watermarks” indicates there is water on the surface of the base metal.

### 3.2. Design of ACGAN Model

In the field of deep learning, the dependence of neural network algorithm on the amount of data is well known. As a prior knowledge, the larger the capacity of the original data, the more sufficient the information about the target it contains, which is beneficial to the training of deep neural network. However, in some experimental scenarios where it is difficult to measure or the experimental measurement data is very complicated, it is hard to collect the data. In order to solve this problem, we can use the data enhancement ability of ACGAN to generate fake data and expand the data capacity for training deep neural network.

The Pytorch deep learning framework (Pytoch is the python version of torch (version 1.6.0), which is an open-source neural network framework by Facebook. The pytoch used in this article is downloaded from the Internet.) is used in all of the experiments. The Anaconda3 virtual environment is used for management. The Python version is 3.6.12, the optimizer is “Adam”, and the learning rate is 0.001. The detailed configurations of the experimental environment can be found in Table 1.

During the experiment, we designed a model structure of ACGAN for defect detection of laser welding data. The structure of ACGAN is shown in Table 2 and Table 3. In designing the structure of generator and discriminator, Leaky ReLu is selected as the activation function of each layer through comparative experiments. Such a choice also solves the problem of neuron death. Dropout is used after each convolution layer, which can eliminate the joint adaptability between neurons and enhance the generalization ability of neural network. Using the structure described above, generator G and discriminator D can generally be optimized after 120 training epochs.

### 3.3. ACGAN-SVM-CNN Defect Detection Fusion Algorithm

**Figure 4:**
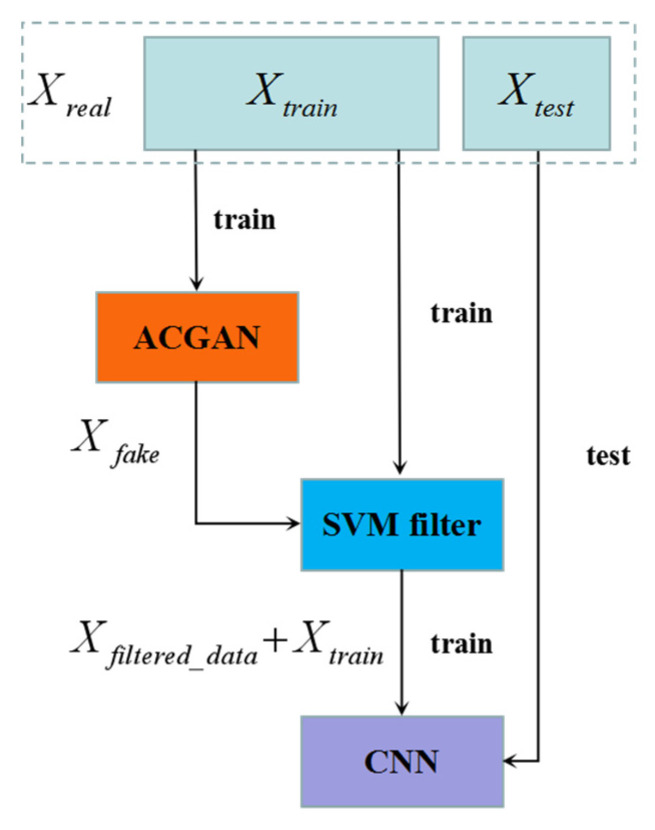
ACGAN-SVM-CNN.

The training difficulty of GAN is usually greater than that of a general neural network. When D and G are entangled with each other, the generator generated low-quality and high-quality data. Therefore, the fake data $X_{fake}$ generated by the generator of the ACGAN algorithm model are not all optimal data. In order to eliminate some inferior fake data, we proposed to use original training data $X_{train}$ to train a filtering model based on SVM algorithm, so as to screen the generated fake data, and combined the filtered data $X_{filtered\_data}$ and training data $X_{train}$ together for the training of CNN algorithm model. In order to compare the performance of the fusion algorithm, the data of the test set was derived from real data, and the test set was always kept unchanged. The fused algorithm model is shown in Figure 4.

### 3.4. Data Filtering and Purification Strategy

**Figure 5:**
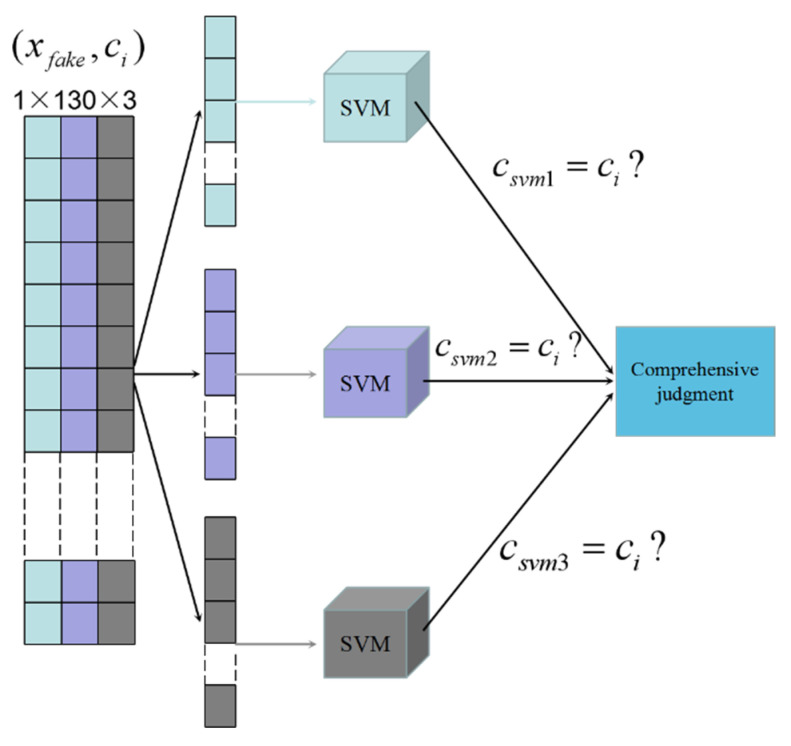
Integrated decision model.

Our goal is to select data which can approximate the original data distribution characteristics from the generated data. Using the ACGAN model designed in this paper to generate 2010 pieces of data (201 pieces of data are generated for each welding defect), and the classification accuracy of ACGAN discriminator is 85.13%. For the generated fake data, it is not always very close to the original data distribution, and those inferior data need to be filtered out. In this issue, we propose to use the concept of ensemble learning to filter data. We use the original real data (6467 × 1 × 130 × 3) to train three SVM models, which can be used to filter the fake data. Three SVM classifiers are trained by using the three features in the original data, so that each feature data can be trained to obtain a classifier. In the ensemble learning theory, a strong learner can be formed by training several individual learners and following certain combination strategies [49,50]. We collected three physical quantities (plasma intensity, light intensity and temperature) in the welding process to determine if there is a welding defect or not. These classifiers are then used to identify whether each feature in the generated fake data corresponds to the type of welding defect. The features of the generated fake data are input into the corresponding SVM classifier for classification. If two or more classifiers give correct classification results, the fake data can be retained. The process is shown in Figure 5.

Through the above filtering method, 2010 pieces of data were filtered and 1147 pieces of data were obtained. In this way, the original data were expanded from 6467 pieces to 7614 pieces. By eliminating the fake data which does not match the original data distribution, the differences between categories will be more prominent, which lays a good foundation for the subsequent training of the classification model.

### 3.5. Comparative Experiment and Result Analysis

In order to compare with the traditional CNN method, we divide the original data into two parts, 5174 pieces are taken as the general training set and 1293 pieces are taken as the general test set. When dividing data sets, the balance of each category of data is fully considered. The fake data filtered by ACGAN- SVM ensemble model and 5174 pieces of original data are combined as an enhanced training set. The enhanced training set is sent to the CNN model for training, and then the general test set is used to test the accuracy of CNN.

**Figure 6:**
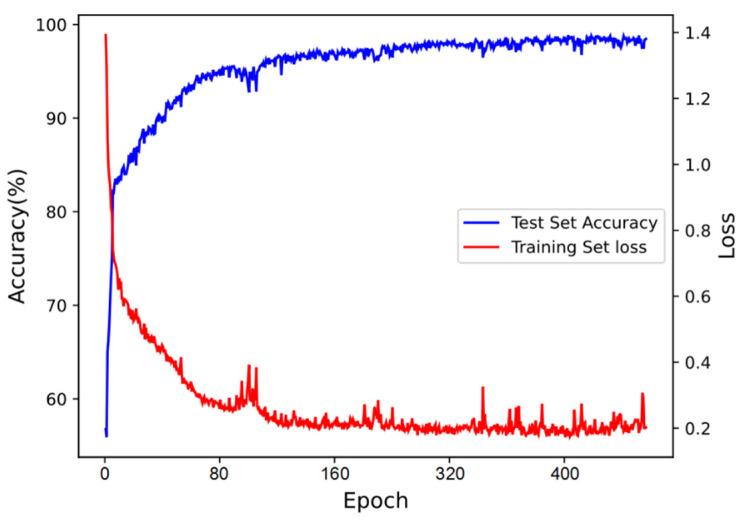
Loss and accuracy curve of ACGAN-SVM-CNN.

The changing curve of loss in the training process and the changing curve of accuracy in the test set are shown in Figure 6. Finally, the classification accuracy of our proposed ensemble model can reach 98.37%.

**Figure 7:**
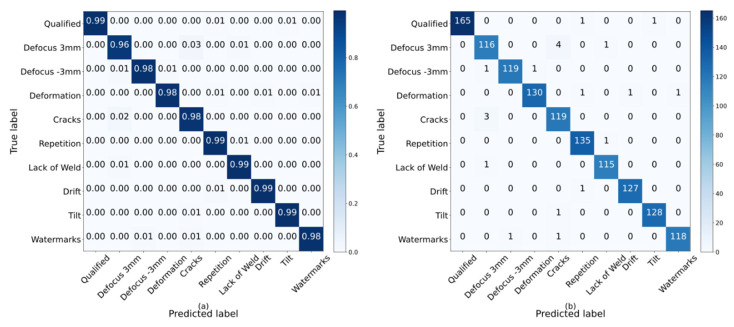
Confusion matrix of ACGAN-SVM-CNN integrated model for welding defect identification. (**a**) Confusion matrix of the classification result; (**b**) confusion probability matrix of the classification result.

Figure 7 shows the confusion matrix obtained from 10-fold cross-validation on the enhanced dataset. The analysis of the confusion matrix shows that the CNN classifier at the final training site has a good classification effect. However, there are some deficiencies in the distinction between ”Defocus 3 mm“ and “Cracks”. This indicates that our classifier is not very good at learning the difference between these two types of welding defects and needs further improvement in future research.

Through Figure 7, it can be seen that the classification prediction result of “Defocus 3 mm” is relatively worse. The model sometimes classifies “Defocus 3 mm” into “Cracks” by mistake, which indicates that the two welding defect states have high similarity. As shown in Table 4, we use precision, recall and F1 (F1 score) to measure the detection performance of the model in different categories. The abbreviations “Qua”, “Def3”, “Def-3”, “Defor”, “Cra”, “Rep”, “LoW”, “Dri”, “Tilt” and “W” in the table represent “Qualified”, “Defocus 3 mm”, “Defocus −3 mm”, “Deformation”, “Cracks”, “Repetition”, “Lack of Weld”, “Drift”, “Tilt” and “Watermarks”, respectively. It can be seen from Table 4 that our proposed fusion model has the best performance in detecting the “Qualified” state, but the recognition of “Defocus 3 mm” category is relatively inferior.

The diversity of samples is enriched through data enhancement, and the difference between classes is ensured through filtering and purification of the generated data. In order to compare the time consumption of models with different complexity, we take the training set and test set of the same volume for the timing test (5174 pieces of training data and 1293 pieces of test data), and the results are shown in Table 5. The ACGAN-SVM-CNN can reach an average speed of 0.76 ms per sample, which meets the real-time requirement for industrial production. Because of the complexity of training ACGAN, the training time is relatively long.

In order to further compare the proposed method of ACGAN-SVM-CNN in this paper with other classical methods in machine learning, we adjust the number of original data. We compare the classification accuracy of various algorithm models through reducing the amount of original data by 10%, 20%, 25% and 30%. Table 6 shows the comparison results of classification accuracies of various methods in the case of different amounts of original data. By comparing the experimental results under different data volumes, our proposed detection model has better discrimination ability in detecting defect categories. When the amount of data is reduced to only 75% of the original data, the classification accuracies of all classifiers will decline greatly. In the process of reducing the amount of original data, the accuracy of the proposed fusion model (ACGAN-SVM-CNN) also decays, but it is the slowest one, which reflects the resistance of the model to data loss.

## 4. Conclusions

In this paper, we have proposed an intelligent diagnosis method (ACGAN-SVM-CNN) for detecting laser welding defects. This detection scheme combines and makes full use of the data enhancement ability of ACGAN and the classification ability of CNN. The trained ACGAN model learns the potential space corresponding to all kinds of welding defect data, and generates fake data for data enhancement. In addition, we propose an ensemble learning-based data filtering and data purification method to filter out the bad ones from generated fake data, which makes the difference between the class characteristics of the enhanced dataset more obvious. The filtered enhanced dataset can train models with higher classification recognition rate. This method has been compared with other existing detection models, in which the amount of original training data has been adjusted differently. The experimental results show that the ACGAN-SVM-CNN scheme can detect the categories of welding defects better when the amount of original training data is decreasing. In our further research, we will develop more effective multi-model fusion strategies and model parameter sharing schemes to improve the training speed and recognition ability of the fusion models.

This method has a good reference significance for the industrial sector where it is difficult to obtain process data, and can be easily extended to deal with the fault or defect detection problems of some key machine components.

## Figures and Tables

**Table 1:** Server configuration information.

Parameters	Specifications
RAM	256 G
CPU	Intel(R) Xeon(R) CPU E5-2680 v2 @2.80 GHZ
GPU	GeForce GTX TITAN X
OS	Windows 10

**Table 2:** Structure of the generator.

Operation	Kernel	Strides	Feature Maps	BN?	Dropout	Nonlinearity
input (128 × 512 × 1 × 1)						
Linear	N/A	N/A	128 × 256 × 1 × 1	×	0.0	Leaky ReLu
Linear	N/A	N/A	128 × 16,640 × 1 × 1	×	0.0	N/A
Upsample	scale factor = 2		128 × 128 × 130 × 4			
Convolution	3 × 2	1 × 1	128 × 128 × 130 × 3	√	0.0	Leaky ReLu
Convolution	3 × 1	1 × 1	128 × 64 × 130 × 3	√	0.0	Leaky ReLu
Convolution	3 × 1	1 × 1	128 × 1 × 130 × 3	×	0.0	Sigmoid

**Table 3:** Structure of the discriminator.

Operation	Kernel	Strides	Feature Maps	BN?	Dropout	Nonlinearity
input (1 × 130 × 3)						
Convolution	3 × 3	2	128 × 16 × 65 × 2	×	0.2	Leaky ReLu
Convolution	3 × 3	2	128 × 32 × 33 × 1	√	0.2	Leaky ReLu
Convolution	3 × 3	2	128 × 32 × 17 × 1	√	0.2	Leaky ReLu
Convolution	3 × 3	2	128 × 64 × 9 × 1	√	0.2	Leaky ReLu
Convolution	3 × 3	2	128 × 128 × 5 × 1	√	0.2	Leaky ReLu

**Table 4:** Performance metrics of ACGAN-SVM-CNN integrated model in each category.

	Class	Qua (%)	Def3 (%)	Def-3 (%)	Defor (%)	Cra (%)	Rep (%)	LoW (%)	Dri (%)	Tilt (%)	W (%)
Metrics											
Precision		100.0	95.87	99.17	99.24	95.20	97.83	98.29	99.22	99.22	99.16
Recall		98.80	95.87	98.35	97.74	97.54	99.26	99.14	99.22	99.22	98.33
F1		99.40	95.87	98.75	98.48	96.35	98.54	98.71	99.22	99.22	98.74

**Table 5:** Training and testing time of different models.

	Training (min)	Testing (ms/Sample)
ACGAN-SVM-CNN	21.61	0.76
ACGAN-CNN	16.19	0.71
CNN	6.81	0.69
SVM	4.53	1.78
ADABOOST	5.21	2.26

**Table 6:** Performance of different models under different amount of training data.

	100%	90%	80%	75%	70%
ACGAN-SVM-CNN	98.37%	98.02%	96.78%	92.02%	91.15%
ACGAN-CNN	97.86%	97.33%	96.62%	92.18%	90.04%
CNN	96.83%	96.17%	95.82%	86.75%	86.33%
ACGAN discriminator	85.13%	76.54%	64.85%	60.37%	53.26%
SVM	83.35%	81.47%	80.26%	65.31%	64.25%
ADABOOST	81.65%	80.25%	71.35%	66.27%	63.93%

## Data Availability

The data presented in this study are available on request from the corresponding author.
